# Chronic cocaine induces HIF-VEGF pathway activation along with angiogenesis in the brain

**DOI:** 10.1371/journal.pone.0175499

**Published:** 2017-04-27

**Authors:** Wei Yin, Kevin Clare, Qiujia Zhang, Nora D. Volkow, Congwu Du

**Affiliations:** 1 Biomedical Engineering Department, Stony Brook University, Stony Brook, NY, United States of America; 2 National Institute on Alcohol Abuse and Alcoholism, National Institutes of Health, Bethesda, MD, United States of America; National University of Singapore, SINGAPORE

## Abstract

Cocaine induces vasoconstriction in cerebral vessels, which with repeated use can result in transient ischemic attacks and cerebral strokes. However, the neuroadaptations that follow cocaine’s vasoconstricting effects are not well understood. Here, we investigated the effects of chronic cocaine exposure (2 and 4 weeks) on markers of vascular function and morphology in the rat brain. For this purpose we measured nitric oxide (NO) concentration in plasma, brain neuronal nitric oxide synthase (nNOS or NOS1), HIF-1α, and VEGF expression in different brain regions, i.e., middle prefrontal cortex, somatosensory cortex, nucleus accumbens, and dorsal striatum, using ELISA or Western blot. Additionally, microvascular density in these brain regions was measured using immunofluorescence microscopy. We showed that chronic cocaine significantly affected NOS1, HIF-1α and VEGF expression, in a region- and cocaine treatment-time- dependent manner. Cerebral microvascular density increased significantly in parallel to these neurochemical changes. Furthermore, significant correlations were detected between VEGF expression and microvascular density in cortical regions (middle prefrontal cortex and somatosensory cortex), but not in striatal regions (nucleus accumbens and dorsal striatum). These results suggest that following chronic cocaine use, as cerebral ischemia developed, NOS1, the regulatory protein to counteract blood vessel constriction, was upregulated; meanwhile, the HIF-VEGF pathway was activated to increase microvascular density (i.e., angiogenesis) and thus restore local blood flow and oxygen supply. These physiological responses were triggered presumably as an adaptation to minimize ischemic injury caused by cocaine. Therefore, effectively promoting such physiological responses may provide novel and effective therapeutic solutions to treat cocaine-induced cerebral ischemia and stroke.

## Introduction

Cocaine is a commonly used illicit substance, the abuse of which is associated with cerebral vascular pathology including transient ischemic attacks (TIA) and cerebral strokes [1]. Even though the mechanism of cocaine-related TIA and stroke are not completely understood, it is generally accepted that cocaine-induced vasoconstriction is a key contributing factor [2]. The effects of cocaine in various brain regions differ, not only because it causes increases in dopamine release and metabolism [3], but also due to cocaine-induced cerebral vasospasm, which appears to be most prominent in frontal cortical regions including prefrontal cortex and somatosensory cortex [4], where cerebral blood flow is most affected [5, 6]. Indeed, using contrast-enhanced ultrahigh-resolution optical coherence tomography, we recently showed that single or repeated cocaine administration to the mouse somatosensory cortex induced cerebral micro-ischemia [7].

Several neuroadaptations have been reported following cerebral ischemia. Reduced cerebral blood flow (CBF) and hypoxic conditions can activate the nitric oxide (NO) signaling pathway [8, 9]. NO, produced by nitric oxide synthase (NOS), relaxes blood vessel wall smooth muscle cells, resulting in vasodilation. Cocaine has been reported to cause vascular endothelial cell dysfunction [10, 11] and interfere with the synthesis and/or release of NO [12].

Angiogenesis has also been observed in areas adjacent to brain ischemia, as a physiological response to enhance local blood flow and oxygen supply [13]. Vascular endothelial growth factor (VEGF) plays a critical role in cerebral angiogenesis [14]. It was reported that both brain injury and hypoxic conditions can promote VEGF expression in brain tissue, leading to the formation of new blood vessels [15–17]. Hypoxic conditions can also lead to the expression of Hypoxia Inducible Factor 1 (HIF-1) [18, 19], which regulates VEGF transcription. Studies conducted by Marti *et al*. demonstrated that HIF-VEGF signal transduction pathway was likely involved in angiogenesis following cerebral ischemia [20]. An *in vitro* study in cultured rat glial cells demonstrated that cocaine treatment led to increased expression of both VEGF and HIF-1 [21]. We also recently demonstrated that chronic cocaine treatment induced a significant increase in microvascular density and VEGF expression in somatosensory cortex in rats [22]. However, the mechanisms involved in the brain responses to cocaine-induced ischemia are poorly understood. The role of the HIF-VEGF signal transduction pathway is not clear, and there have been no studies assessing brain regional differences in the neuroadaptations that follow cocaine exposures.

To address this neglect, we investigated the effects of chronic cocaine exposure on neurochemical markers associated with ischemia and microvasculature density in the rat brain. Specifically, we assessed the effects of chronic cocaine on NO release, Nitric Oxide Synthase 1 (NOS1), HIF-1 and VEGF expression, as well as microvascular density in brain regions with high sensitivity to cocaine’s effects including cortical (e.g., middle prefrontal cortex and somatosensory cortex) and striatal regions (e.g., nucleus accumbens and dorsal striatum).

## Materials and methods

### Animal preparation

Adult Sprague-Dawley male rats (14 weeks of age, 250–300g, from Taconic) were treated daily with cocaine (30 mg/kg) by intraperitoneal injection for 2 or 4 weeks [22]. Sham animals were treated with 0.9% saline (0.7 mL/100 g) instead. Some rats were used for brain imaging, as reported previously [22]. For immunofluorescence microscopy of the rat brain vasculature, after imaging experiments, 500 μL FITC-Dextran (MW 2,000 KDa, at 50 mg/mL, from Sigma-Aldrich) was injected into the rat left ventricle and let circulate for 1 minute before euthanasia. Rat brain was then extracted and fixed with 4% formaldehyde overnight at 4°C. For plasma nitric oxide (NO) concentration measurement, rats were not perfused with FITC-Dextran. Instead, 1–2 mL of whole blood was collected (anti-coagulated with 0.32% sodium citrate) by cardiac puncture. For all the other experiments (i.e., NOS1, VEGF and HIF-1α expression measurements), rat brain was dissected directly (no cardiac puncture for blood collection, and no perfusion with FITC-Dextran). Brain samples from different regions, including middle prefrontal cortex (mPFC), somatosensory cortex (Ssc), nucleus accumbens (NAc), and dorsal striatum (DStr), were placed into tissue extraction reagent (Thermo Fisher Scientific) containing protease and phosphatase inhibitors. Collected samples were then homogenized at 15,000 Hz for 15 seconds using a tissue homogenizer (Tissue Master 125, OMNI International Inc.). After incubation on ice for 1 hour, samples were centrifuged at 14,500*×g* for 20 min (4°C). Supernatant was collected and stored at -80°C until use.

All animals used in this study were housed in the Division of Laboratory Animal Resources (DLAR) of Stony Brook University before experiments. Animals were housed in pairs, maintained in a 12h:12h-light:dark cycle, and provided with fresh water and normal diet. All the procedures reported in this study were performed on anesthetized animals. Isoflurane was used for surgical plane anesthesia and before perfusion, so the animals would not perceive any pain during the experiments. At the end of the experiments, animals were euthanized with an anesthetic overdose. 3–4 rats were used for each control group (2 weeks or 4 weeks); 3–9 rats were used for each cocaine-treatment (2 weeks and 4 weeks) group. Detailed numbers of animals used for each experiment are reported in the Results section.

#### Ethics statement

This study was carried out in strict accordance with the recommendations in the Guide for the Care and Use of Laboratory Animals of the National Institutes of Health. The protocol was approved by the Institutional Animal Care and Use Committee of Stony Brook University (IRBNet: 252462). The animals were anesthetized and all efforts were made to minimize suffering.

### Plasma nitric oxide (NO) concentration

Following centrifugation (1000*×g*, 9 minutes) of whole rat blood, platelet poor plasma was collected and assayed for NO concentration using a commercial Nitric Oxide Assay kit (Abcam). In this assay, plasma total nitrate concentration was measured, which was used to calculate NO production, as NO produced *in vivo* was rapidly oxidized to nitrate.

### Nitric oxide synthase 1 (NOS1) expression

NOS1 expression in rat brain tissue was measured using an ELISA kit (Abcam). Briefly, extracted brain tissue homogenates were placed on microtiter plates, followed by addition of NOS1 capture antibody and detection antibody. Bound antibody was detected using a TMB substrate (Tetramethylbenzidine) and read at 450 nm in a microplate reader (SpectraMax i3, Molecular Devices, Sunnyvale, CA).

### Brain microvascular density quantification

Formaldehyde-fixed brain tissue was equilibrated in 30% sucrose solution overnight at 4°C. Tissue samples were then put in OCT medium (Thermo Fisher Scientific) and let freeze at -80°C, before slicing (8–10 μm) using a cryostat (Leica CM3050S, Leica Biosystems, Richmond, IL). Microvasculature in the rat brain tissue was visualized using a Nikon E80i fluorescence microscope, and microvascular density was quantified by counting the number of fluorescent vessels per unit area (from FITC-Dextran) using the *Image J* software [22]. Brain samples that were not perfused with FITC-Dextran were used as the negative control, to determine the background fluorescence intensity.

### VEGF expression

Freshly collected rat brain tissue was fixed in 4% formaldehyde, followed by equilibration in 30% sucrose solution. Sliced tissue samples (~ 8 μm) were blocked in blocking buffer for 30 minutes before a polyclonal rabbit anti-rat VEGF antibody (Abcam) was added. Primary antibody binding was detected using an Alexa Fluor 488 conjugated goat anti-rabbit secondary antibody (Jackson ImmunoResearch). VEGF expression in rat brain tissue was quantified by intensity analysis using the *Image J* software [22].

To visualize the co-localization of micro-vessels and VEGF expression, some of the brain tissue samples from the FITC-Dextran-perfused rats were incubated with rabbit anti-rat VEGF antibody, followed by detection with Alexa Fluor 594-conjugated anti-rabbit secondary antibody (Jackson ImmunoResearch). DAPI was used to stain cell nuclei, before immunofluorescence microscopy.

### HIF-1α expression

Gel electrophoresis was conducted to separate proteins in rat brain homogenates, followed by Western blot to measure HIF-1α expression, using a polyclonal rabbit anti-rat HIF-1α antibody (1μg/mL, from Abcam). Primary antibody binding was detected using a Europium-labeled goat anti-rabbit secondary antibody (Molecular Devices). Expressed protein bands were detected and analyzed using a SpectraMax i3 reader (Molecular Devices).

### Statistical analysis

Data analysis was conducted using SAS Statistical Software (9.3) or Microsoft Excel. In SAS, one-way ANOVA was used to compare plasma NO concentrations between different experimental conditions, and two-way ANOVA was used to determine how cocaine treatment affected brain microvascular density, NOS1, VEGF, and HIF-1α expression, in different brain regions (i.e., mPFC, Ssc, NAc, and DStr), and if the effect was treatment time-dependent (2 weeks or 4 weeks). To determine if there was a linear correlation between brain tissue VEGF expression and microvascular density, the Pearson product-moment correlation coefficients were calculated using Excel. Significant difference was defined as *P* < 0.05.

## Results

### Chronic cocaine treatment did not change plasma NO level

**Fig 1:**
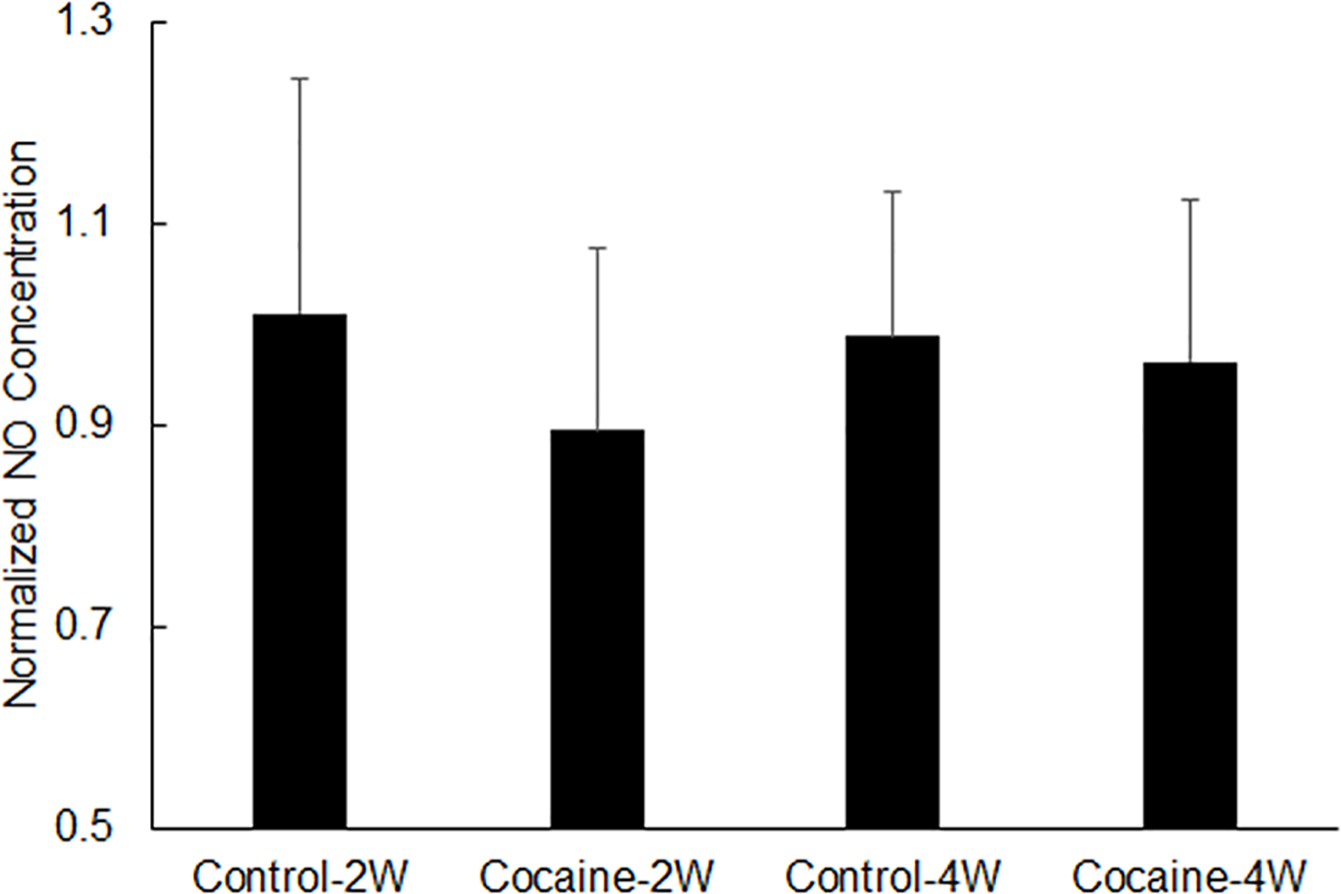
Normalized plasma NO concentration in control rats and cocaine-treated rats. “2W” stands for “2 weeks”, and “4W” stands for “4 weeks”. All data was normalized to the average plasma NO concentration of 2-week control rats. Data is presented as mean + standard error (n = 4–9).

Rat whole blood was collected by cardiac puncture following 2-week or 4-week cocaine (or saline for control rats) treatment. Plasma NO level was measured using a commercial kit. As depicted in Fig 1, even though there was a noticeable decrease in plasma NO concentration (approximately 10%) following 2-week cocaine treatment, no significant difference (n = 4–9, *P* > 0.5) was detected in rat plasma NO levels, between control rats and rats treated with cocaine (2 weeks and 4 weeks).

### Chronic cocaine treatment increased NOS1 expression in the brain

**Fig 2:**
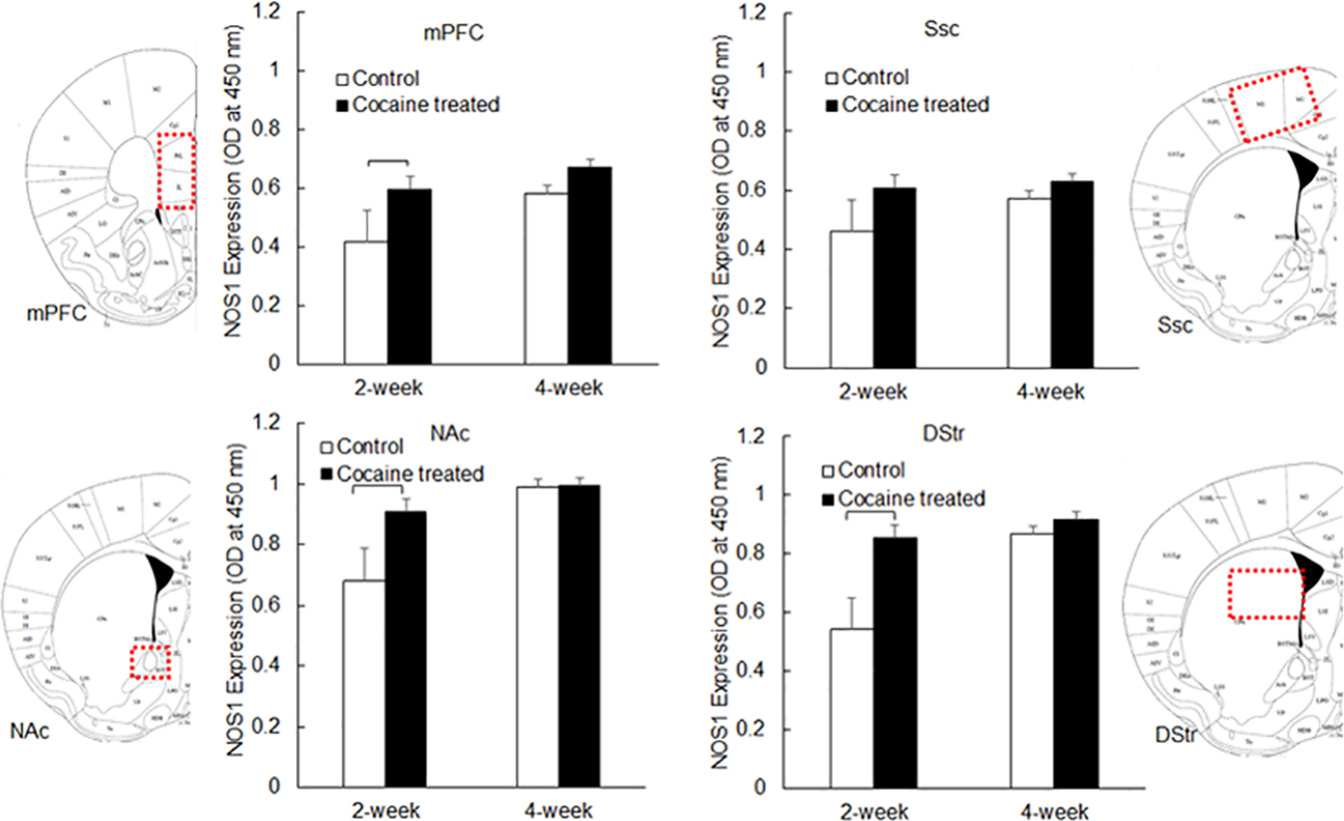
NOS1 expression in rat brain tissue, following 2-week or 4-week cocaine treatment. Data is presented as mean + standard error (n = 3–6). Two-way ANOVA was used to detect significant difference between treatment groups. All significant differences (*P*<0.05) are marked.

Rat brain tissues collected from different brain regions, including mPFC, Ssc, NAc, and DStr, were homogenized before NOS1 expression measurement using a sandwich ELISA. Fig 2 shows the comparison of NOS1 expression between the control and chronic cocaine-treated animals in these brain regions. The results demonstrated that cocaine treatment had a significant effect (*P*<0.0001) on NOS1 expression, and this effect varied significantly among different brain regions. As summarized in Table 1, NOS1 expression was very similar in mPFC and Ssc (*P* = 0.9615). Two-week cocaine treatment increased NOS1 expression in mPFC from 0.42±0.21 (O.D. at 405 nm, mean±standard deviation, n = 4) to 0.60±0.11 (n = 6), which was further increased to 0.67±0.07 (n = 6) after 4-week cocaine treatment. In Ssc, NOS1 expression increased from 0.46±0.19 (n = 4, O.D. at 405 nm, mean±standard deviation) to 0.61±0.19 (n = 6) following 2-week cocaine treatment, and to 0.63±0.09 (n = 6) following 4-week cocaine treatment. These values were significantly different from NOS1 expression in NAc (*P*<0.0001) and DStr (*P*<0.0001). NOS1 expression in NAc increased from 0.68±0.18 (n = 3, O.D. at 405 nm, mean±standard deviation) to 0.91±0.06 (n = 5) following 2-week cocaine treatment. However, 4-week cocaine treatment (0.99±0.11, n = 5) did not cause significant changes in NOS1 expression compared to 4-week control rats (0.99±0.11, n = 3). In DStr, 2-week cocaine treatment significantly increased NOS1 expression from 0.54±0.20 (n = 4) to 0.85±0.21 (n = 6, *P* = 0.049). No significant difference (*P* = 0.5635) was detected between control rats and cocaine treated rats at 4 weeks, as NOS1 expression in DStr increased in both 4-week control rats (0.87±0.06, n = 4) and 4-week cocaine-treated rats (0.92±0.13, n = 6). Two-way ANOVA indicated that cocaine-treated rats (2 weeks and 4 weeks) had a significant increase in NOS1 expression, compared to 2-week control rats (Table 1). Specifically, 2-week cocaine treatment induced significant increases in NOS1 expression in mPFC (*P* = 0.0495), NAc (*P* = 0.0279) and DStr (*P* = 0.0009), but not in Ssc (*P* = 0.1202). No significant differences were detected between 2-week cocaine-treated rats and 4-week cocaine-treated rats (*P* = 0.1308). However, significant increases in NOS1 expression were also observed in 4-week control rats, when compared to 2-week control rats (Table 1).

**Table 1:** NOS1 expression (mean±Std) in different brain regions including mPFC, Ssc, NAc and DStr, following cocaine treatment for 2 weeks and 4 weeks. *P* values calculated using two-way ANOVA (SAS) are listed below. Significant *P* values (*P*<0.05) are bolded.

	mPFC	Ssc	NAc	DStr
2 week—Control	0.42±0.21 (n = 4)	0.46±0.19 (n = 4)	0.68±0.18 (n = 3)	0.54±0.20 (n = 4)
2 week—Cocaine	0.60±0.11 (n = 6)	0.61±0.19 (n = 6)	0.91±0.06 (n = 5)	0.85±0.21 (n = 6)
4 week—Control	0.58±0.05 (n = 4)	0.57±0.04 (n = 4)	0.99±0.10 (n = 3)	0.87±0.06 (n = 4)
4 week—Cocaine	0.67±0.07 (n = 6)	0.63±0.09 (n = 6)	0.99±0.10 (n = 5)	0.92±0.13 (n = 6)

mPFC-Ssc	mPFC-NAc	mPFC-DStr	Ssc-NAc	Ssc-DStr	NAc-DStr
*P* = 0.9615	***P*<0.0001**	***P*<0.0001**	***P*<0.0001**	***P*<0.0001**	***P* = 0.0429**
2 week Control — 4 week Control	2 week Control — 2 week Cocaine	2 week Control — 4 week Cocaine	4 week Control — 2 week Cocaine	4 week Control — 4 week Cocaine	2 week Cocaine — 4 week Cocaine
***P*<0.0001**	***P*<0.0001**	***P*<0.0001**	*P* = 0.7966	*P* = 0.2764	*P* = 0.1308

### Cocaine increased microvascular density in cortex within 2 weeks and in NAc within 4 weeks, but not in DStr

**Fig 3:**
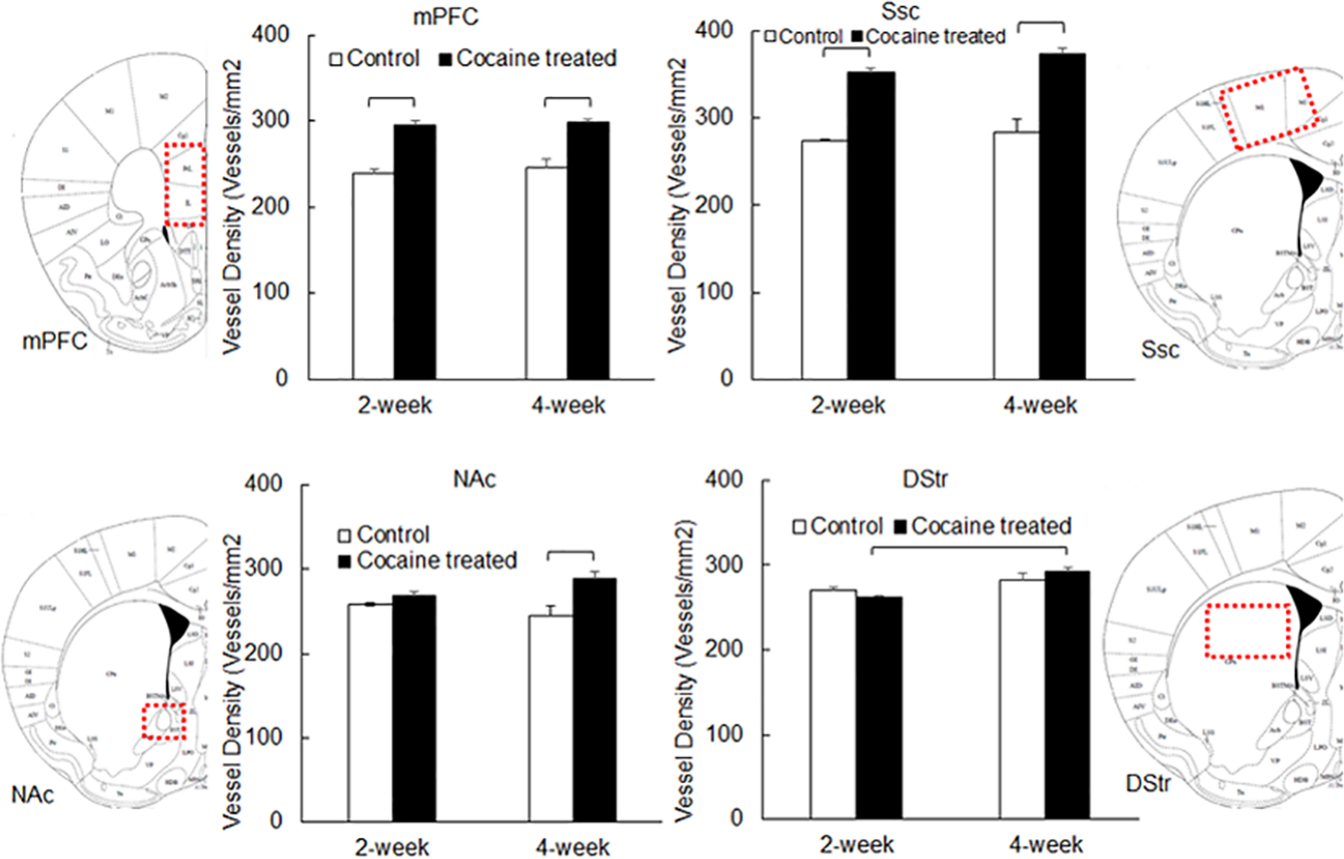
Microvascular density in rat brain tissue measured using immunofluorescence microscopy. Data is presented as mean + standard error (n = 3). ANOVA was used to detect significant difference between treatment groups. All significant differences (*P*<0.05) are marked.

Rat brain tissue slices prepared after FITC-Dextran perfusion were visualized using an inverted Nikon fluorescence microscope. Images were analyzed using the *Image J* software. Fig 3 depicts the quantified microvascular density (number of vessels per unit area) in different brain regions following 2-week or 4-week cocaine (or saline) treatment. Microvascular density varied significantly (*P*<0.0001, Table 2) among different brain regions. Within 2 weeks of cocaine treatment, the microvascular density in both mPFC and Ssc increased significantly (mean ± standard deviation changed from 238.54±8.88 to 295.83±9.19 in mPFC, n = 3, *P*<0.0001; and from 273.73±4.00 to 352.29±8.51 in Ssc, n = 3, *P<0*.*0001*). However, the changes in microvascular density in striatal regions, i.e., NAc and DStr, were not statistically significant (mean ± standard deviation changed from 257.79±4.09 to 268.94±8.88 in NAc, n = 3, *P* = 0.2955; and from 269.86±6.95 to 262.49±2.76 in DStr, n = 3, *P* = 0.4873). However, with 4-week cocaine treatment, increases in microvascular density were not only found in mPFC (from 245.63±17.68 to 298.68±7.48, n = 3, *P*<0.0001) and Ssc (from 283.40±28.08 to 373.38±12.44, n = 3, *P*<0.0001), but also in NAc (from 244.99±20.74 to 287.91±16.61, n = 3, *P* = 0.0003). No significant increase in microvascular density was observed in DStr after 4-week cocaine treatment (from 282.75±12.76 to 292.88±7.31, n = 3, *P* = 0.3411). Statistical analysis (Table 2) indicated that microvascular density was region—and treatment time–dependent (*P*<0.001), and a larger number of new micro-vessels formed in the Ssc, compared to other regions.

**Table 2:** Microvascular density (mean±Std) in different brain regions including mPFC, Ssc, NAc and DStr, following cocaine treatment for 2 weeks and 4 weeks. *P* values calculated using two-way ANOVA (SAS) are listed below. Significant *P* values (*P*<0.05) are bolded.

	mPFC	Ssc	NAc	DStr
2 week—Control	238.5±8.9 (n = 3)	273.7±4.0 (n = 3)	257.8±4.1 (n = 3)	269.9±7.0 (n = 3)
2 week—Cocaine	295.8±9.2 (n = 3)	352.3±8.5 (n = 3)	268.9±8.9 (n = 3)	262.5±2.8 (n = 3)
4 week—Control	245.6±17.7 (n = 3)	283.4±28.1 (n = 3)	245.0±20.7 (n = 3)	282.8±12.8 (n = 3)
4 week—Cocaine	298.7±7.5 (n = 3)	373.4±12.4 (n = 3)	287.9±16.6 (n = 3)	292.9±7.3 (n = 3)

mPFC-Ssc	mPFC-NAc	mPFC-DStr	Ssc-NAc	Ssc-DStr	NAc-DStr
***P*<0.0001**	*P =* 0.37	*P =* 0.1719	***P*<0.0001**	***P*<0.0001**	***P* = 0.0277**
2 week Control — 4 week Control	2 week Control — 2 week Cocaine	2 week Control — 4 week Cocaine	4 week Control — 2 week Cocaine	4 week Control — 4 week Cocaine	2 week Cocaine — 4 week Cocaine
*P =* 0.4273	***P*<0.0001**	***P*<0.0001**	***P*<0.0001**	***P*<0.0001**	***P* = 0.0014**

### Chronic cocaine treatment enhanced VEGF expression in cortex within 2 weeks, and in NAc within 4 weeks, but not in DStr

**Fig 4:**
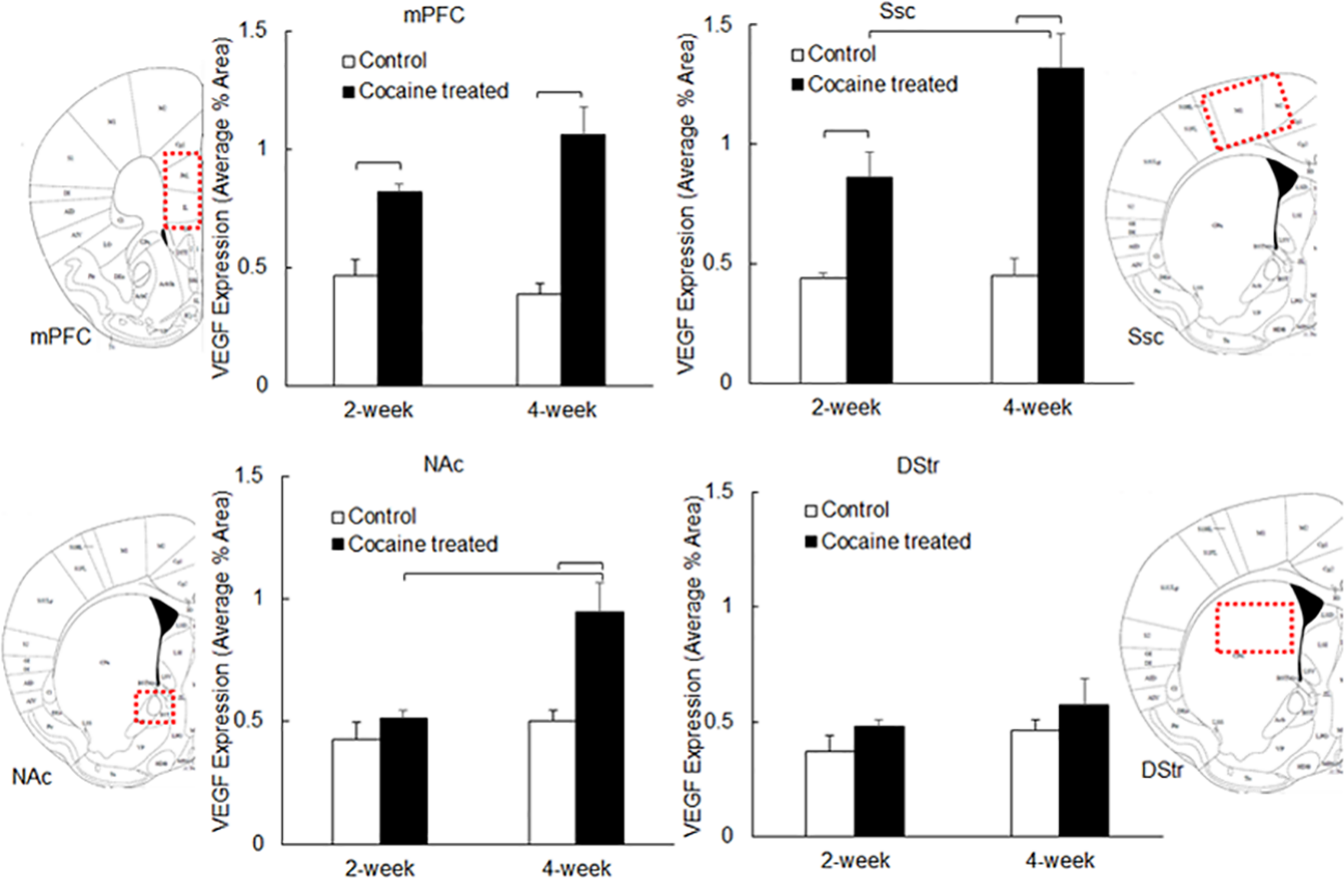
VEGF expression in rat brain tissue measured using immunofluorescence microscopy. Data is presented as mean + standard error (n = 3–4). ANOVA was used to detect significant difference between treatment groups. Significant differences (*P*<0.05) are marked.

Rat brain tissue VEGF expression was also measured using fluorescence microscopy. The results (Fig 4 and Table 3) demonstrated that VEGF expression was also dependent on brain region and treatment time (*P*<0.0001). Specifically, in Ssc, VEGF expression was 0.44±0.05 (mean±standard deviation) in control animals (2 week) and increased to 0.86±21 in animals with 2 weeks of cocaine treatment (n = 3–4, *P* = 0.0024). VEGF expression in mPFC increased from 0.46±12 to 0.82±0.07 (n = 3–4, *P* = 0.0092) after 2-week cocaine treatment. However, no significant changes in VEGF expression were observed in NAc (from 0.43±0.10 to 0.51±0.09, n = 3–4, *P* = 0.5015) or DStr (from 0.37±0.08 to 0.48±0.18, n = 3–4, *P* = 0.4019). Four-week cocaine treatment further enhanced VEGF expression in mPFC (mean±standard deviation: 0.39±0.08 to 1.06±0.24, n = 3–4, *P*<0.0001), Ssc (0.45±0.13 to 1.32±0.30, n = 3–4, *P*<0.0001) and NAc (from 0.50±0.26 to 0.95±0.11, n = 3–4, *P* = 0.0016). Increases in VEGF expression in DStr (0.46±0.17 to 0.57±0.21, n = 3–4) were not significant (*P* = 0.4058). Regarding regional differences (Table 3), VEGF expression in Ssc was significantly higher than that in NAc and DStr (*P* = 0.0138 and *P*<0.0001 respectively), and VEGF expression in mPFC was significantly higher than that in DStr (*P* = 0.0025).

**Table 3:** VEGF expression (mean±Std) in different brain regions including mPFC, Ssc, NAc and DStr, following cocaine treatment for 2 weeks and 4 weeks. *P* values calculated using two-way ANOVA (SAS) are listed below. Significant *P* values (*P*<0.05) are bolded.

	mPFC	Ssc	NAc	DStr
2 week—Control	0.46±0.12 (n = 3)	0.44±0.05 (n = 3)	0.43±0.10 (n = 3)	0.37±0.08 (n = 3)
2 week—Cocaine	0.82±0.07 (n = 4)	0.86±0.21 (n = 4)	0.51±0.09 (n = 4)	0.48±0.18 (n = 4)
4 week—Control	0.39±0.08 (n = 3)	0.45±0.13 (n = 3)	0.50±0.26 (n = 3)	0.46±0.17 (n = 3)
4 week—Cocaine	1.06±0.24 (n = 4)	1.32±0.30 (n = 4)	0.95±0.11 (n = 4)	0.57±0.21 (n = 4)

mPFC-Ssc	mPFC-NAc	mPFC-DStr	Ssc-NAc	Ssc-DStr	NAc-DStr
*P* = 0.2101	*P* = 0.2008	***P* = 0.0025**	***P = 0*.*0138***	***P*<0.0001**	*P* = 0.0621
2 week Control — 4 week Control	2 week Control — 2 week Cocaine	2 week Control — 4 week Cocaine	4 week Control — 2 week Cocaine	4 week Control — 4 week Cocaine	2 week Cocaine — 4 week Cocaine
*P = 0*.*7063*	***P = 0*.*0006***	***P*<0.0001**	***P* = 0.0018**	***P<0*.*0001***	***P*<0.0001**

### Increase in microvascular density correlated to changes of VEGF expression in cortex, but not in striatum

**Fig 5:**
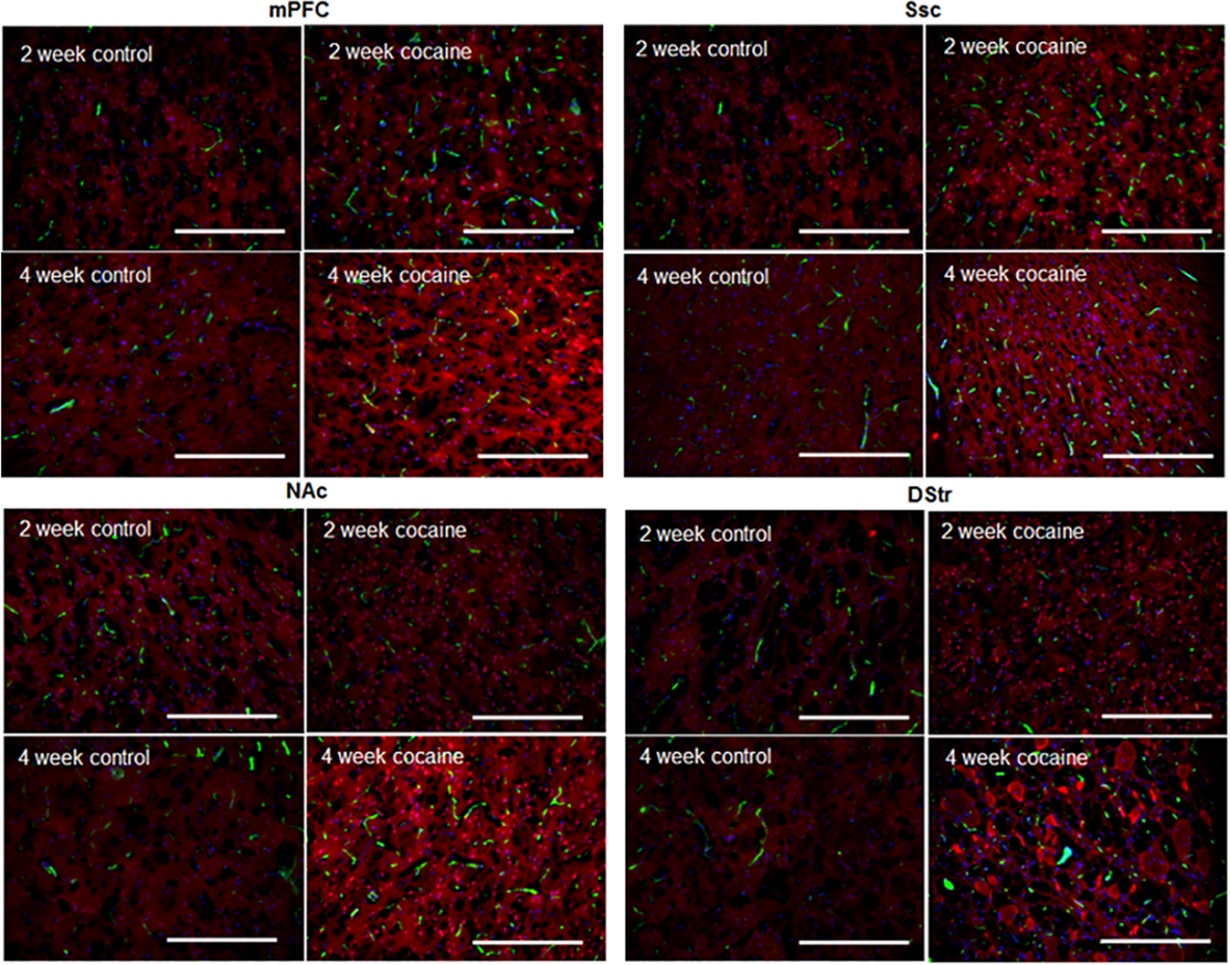
Representative images of VEGF and microvasculature co-localization within different brain regions. Scale bars stand for 100 μm.

**Fig 6:**
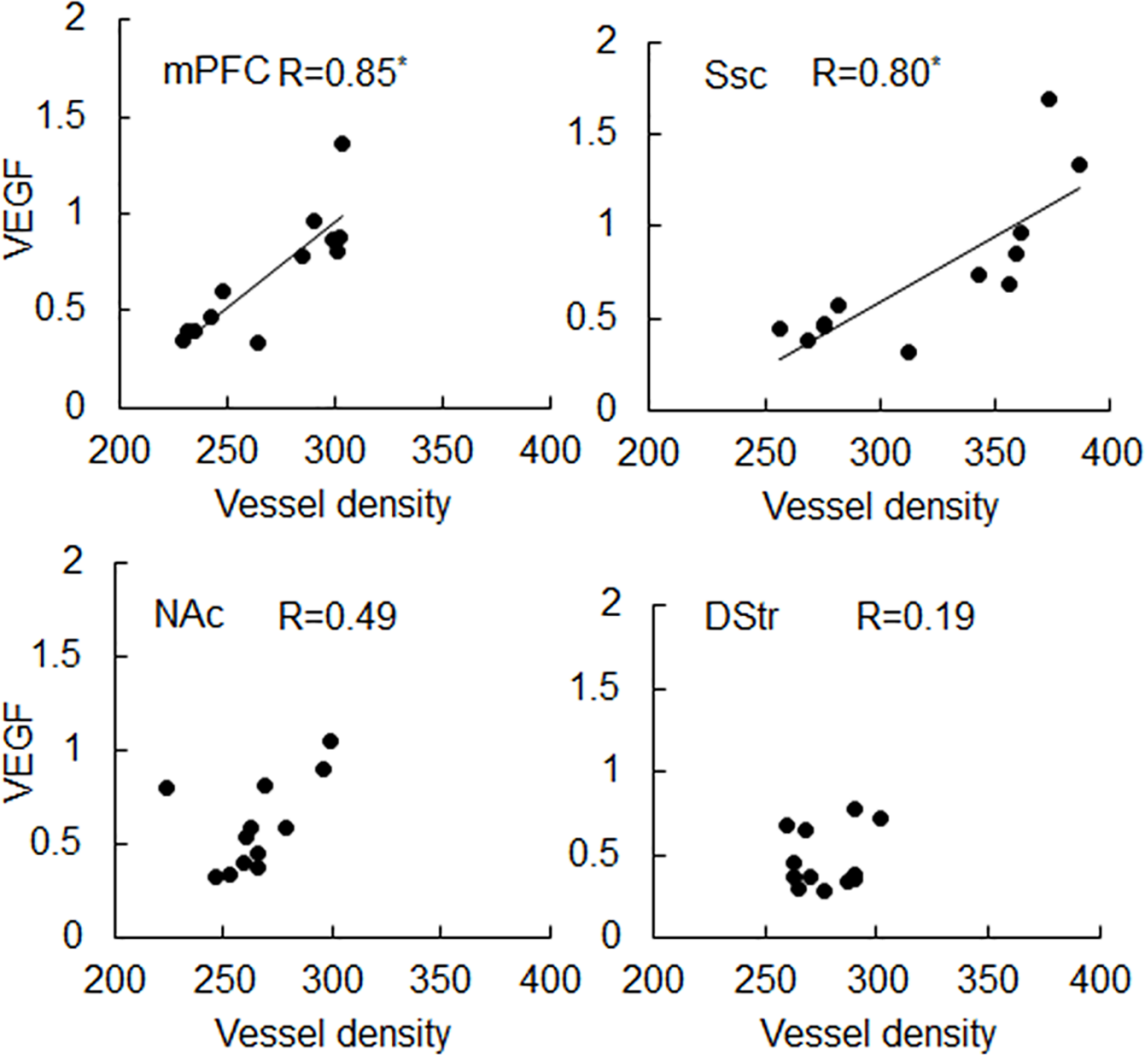
Correlations between VEGF expression and microvascular density in the middle prefrontal cortex (mPFC), the somatosensory cortex (Ssc), nucleus accumbens (NAc) and dorsal striatum (DStr). The Pearson product-moment correlation coefficients (R) are listed on each panel. Significant (*P*<0.05) R values are marked with *.

Representative images of micro blood vessels and expressed VEGF in different brain regions are depicted in Fig 5, demonstrating the co-localization of microvasculature and VEGF expression in the brain tissue. Consistent with that shown in Figs 3 and 4, cocaine treatment seemed to have more profound effects on both microvascular (green fluorescence) density and VEGF expression (red fluorescence) in mPFC, (Fig 5, top left panels) and Ssc (Fig 5, top right panels), compared to striatal regions (NAc and DStr, Fig 5, bottom panels), especially at 2 weeks. Following 4-week cocaine exposure, VEGF expression in both cortical (i.e., mPFC and Ssc) and striatal (i.e., NAc) regions increased significantly, compared to 2-week cocaine treatment. Longer cocaine treatment time (i.e., 4 weeks) induced a visible increase in microvascular density in NAc (compared to 2 weeks), but not in DStr. The correlations between tissue VEGF expression and microvascular density in different brain regions are depicted in Fig 6, suggesting that there was a significant correlation between VEGF expression and microvascular density in mPFC (R = 0.85, *P*<0.001) and Ssc (R = 0.80, *P*<0.01), but not in NAc (R = 0.49, *P*>0.1) or DStr (R = 0.19, *P*>0.1).

### 4-week cocaine treatment induced significant changes in HIF-1α expression in mPFC

**Fig 7:**
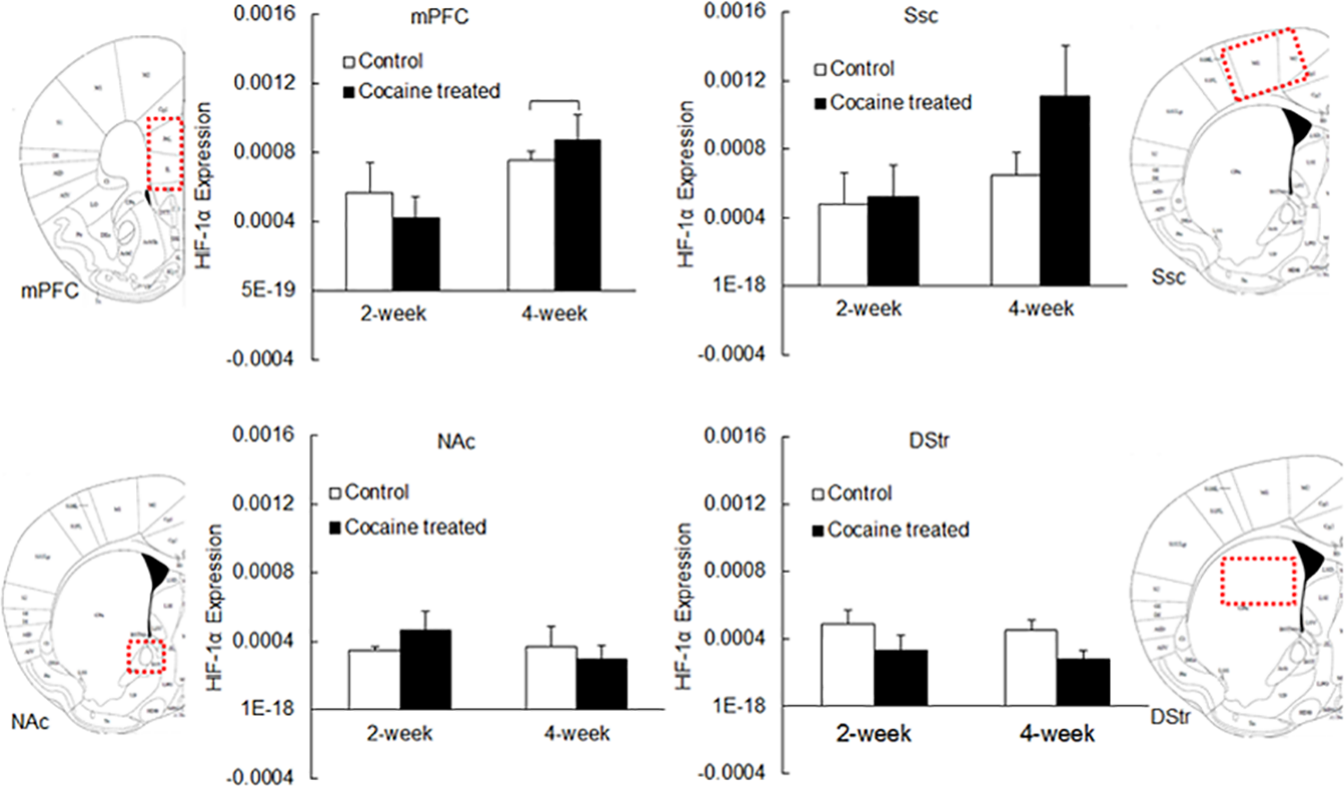
HIF-1α expression in rat brain tissue measured using Western blot. Data is presented as mean + standard error (n = 3–9). ANOVA was used to detect significant difference between treatment groups. Significant differences (*P*<0.05) are marked.

The expression of HIF-1α, the VEGF transcription factor, in different brain regions was measured using Western blot (A representative image of Western blot bands for HIF-1α expression is depicted in S1 Fig). As shown in Table 4, the effect of cocaine on HIF-1α expression was region-dependent. Two-way ANOVA indicated that HIF-1α expression in mPFC was significantly different from that in Ssc, NAc and DStr (*P* values were 0.0316, 0.0025 and 0.0023 respectively). HIF-1α expression in mPFC, Ssc, NAc and DStr following 2-week and 4-week cocaine treatment is depicted in Fig 7. Two-week cocaine exposure did not cause any noticeable changes in HIF-1α expression in any of the brain regions examined (i.e., mPFC, Ssc, NAc, and DStr, *P* = 0.9759). Significant increase in HIF-1α was only detected in mPFC following 4-week cocaine treatment (normalized band intensity of Western blot, presented as mean±standard deviation: from (7.51±0.87)×10^−4^ to (8.72±4.00)×10^−4^, n = 3–8, *P* = 0.0004). Though enhanced HIF-1α was observed in Ssc following 4-week cocaine treatment (from (6.46±2.71)×10^−4^ to (11.12±8.8)×10^−4^, n = 4–9), the differences were not significant (*P* = 0.3609). In NAc and DStr, HIF-1α expression was not affected by 4-week cocaine treatment (NAc: from (3.30±1.67)×10^−4^ to (2.99±1.67)×10^−4^, n = 3–5, *P* = 0.9610; DStr: from (4.02±0.65)×10^−4^ to (2.77±1.49)×10^−4^, n = 3–7, *P* = 0.8307).

**Table 4:** HIF-1α expression (mean±Std) in different brain regions including mPFC, Ssc, NAc and DStr, following cocaine treatment for 2 weeks and 4 weeks. *P* values calculated using two-way ANOVA (SAS) are listed below. Significant *P* values (*P*<0.05) are bolded.

	mPFC (×10^−4^)	Ssc (×10^−4^)	Nac (×10^−4^)	DStr (×10^−4^)
**2 week—Control**	**5.62±3.05 (n = 3)**	**4.81±3.57 (n = 4)**	**3.50±0.37 (n = 3)**	**4.91±1.36 (n = 3)**
**2 week—Cocaine**	**4.21±3.72 (n = 9)**	**5.23±4.80 (n = 7)**	**4.64±2.70 (n = 6)**	**3.34±2.45 (n = 8)**
**4 week—Control**	**7.51±0.87 (n = 3)**	**6.46±2.71 (n = 4)**	**3.30±1.67 (n = 3)**	**4.02±0.65 (n = 3)**
**4 week—Cocaine**	**8.72±4.00 (n = 8)**	**11.1±8.80 (n = 9)**	**2.99±1.67 (n = 5)**	**2.77±1.49 (n = 7)**

mPFC-Ssc	mPFC-NAc	mPFC-DStr	Ssc-NAc	Ssc-DStr	NAc-DStr
***P* = 0.0316**	***P* = 0.0025**	***P* = 0.0023**	***P = 0*.*2465***	***P = 0*.*2552***	***P* = 0.9593**
**2 week Control** **—** **4 week Control**	**2 week Control** **—** **2 week Cocaine**	**2 week Control** **—** **4 week Cocaine**	**4 week Control** **—** **2 week Cocaine**	**4 week Control** **—** **4 week Cocaine**	**2 week Cocaine** **—** **4 week Cocaine**
***P = 0*.*0519***	***P = 0*.*9759***	***P = 0*.*5062***	***P* = 0.0228**	***P = 0*.*1067***	***P = 0*.*3886***

Raw data collected from all the experiments can be found in the S1 Dataset.

## Discussion

In rodent models of chronic cocaine, we recently showed that repeated cocaine exposure caused vasoconstriction and cerebral ischemia [23, 24], which supported the clinical findings of increased risk for TIA and cerebral strokes in cocaine abusers. However, the cellular responses to the cerebral ischemic complications induced by cocaine are not well understood. In the present study, we investigated if chronic use of cocaine could directly affect the physiological response of the cerebral vasculature, cause HIF-VEGF signal transduction pathway activation, and lead to increased microvascular density, or, cerebral angiogenesis, in different brain regions, including cortex (mPFC and Ssc) and straitum (NAc and DStr). Our results demonstrated that cocaine use had significant effects on cerebral tissue NOS1, VEGF, HIF-1α expression, and microvascular density, in a region-dependent and time-dependent manner.

NO is a powerful vasodilator and helps to dilate constricted blood vessels thus enhancing local CBF. Studies by Lee *et al* reported that both acute and repeated cocaine injection in rats significantly increased NO production in DStr. In their studies, they directly measured NO levels within the rat’s brain tissue in real time using highly specialized NO biosensors [25, 26]. Studies have also reported that the activation of neuronal nitric oxide (nNOS, also known as NOS1) enhanced NO production in mPFC and DStr following repeated cocaine administration in rats [27, 28]; and the use of 7-nitroindazole, an NOS1 inhibitor, reduced NO production following chronic cocaine exposure [29]. Few studies had directly measured NOS1 production induced by chronic cocaine treatment. In the present study, both plasma NO concentration and rat brain tissue total NOS1 expression were measured. No significant changes were observed in plasma NO concentration following 2-week or 4-week cocaine treatment (Fig 1), which may have resulted from the low overall NO concentration in blood plasma. Also, measuring blood nitrate concentration (then covert to NO concentration) was not likely the most sensitive way to detect the small and transient changes in plasma NO concentration, compared to specialized NO biosensors developed by others. Significant increases in NOS1 expression within different brain regions (i.e., mPFC, SSc, Nac and DStr) were observed after 2-week or 4-week cocaine treatment, which were consistent with the trend reported by others in NOS1 activation and NO production [27, 28]. These results demonstrated that chronic cocaine use could trigger significant changes in vascular regulatory proteins such as NOS1, to counteract cerebral vasoconstriction induced by cocaine. Interestingly, the level of such a physiological response varied significantly (Table 1) within different brain regions, with the largest changes occurring in striatum, i.e., NAc (increased by 33.8% after 2 week cocaine treatment) and DStr (increased by 57.4% after 2-week cocaine treatment). NOS1 expression also increased in 4-week control rats (increased by 38% in mPFC, 24% in Ssc, 46% in NAc, and 61% in DStr respectively), compared to 2-week controls. We speculate such increases might have been caused by stress induced by daily saline injection for 4 weeks [30]. Based on this data, it’s hard to determine if the increases observed in NOS1 expression in cocaine-treated animals were a direct consequence of stress, or resulted from the combined effect of cocaine and stress.

We also observed that the microvascular density increased significantly in cortical regions including mPFC and Ssc (Fig 3). Two-way ANOVA (Table 2) demonstrated that microvascular density varied significantly within different brain regions; 2-week cocaine treatment induced significant increases in brain microvascular density, which was further enhanced as cocaine treatment time elongated (4 weeks). It is well established that VEGF expression correlates with increased microvascular density after brain ischemia [31–33], suggesting that angiogenesis might be responsible for the increased vascular density following ischemia. Importantly, in the present study, significant increase in VEGF expression was observed in cortex (mPFC and Ssc), but not in striatum (NAc and DStr), following two-week cocaine treatment (Fig 4). Furthermore, cocaine treatment time also had a significant effect on VEGF expression (Table 3). Statistical analysis (Fig 6) suggested that the correlation between VEGF expression and microvascular density within cortical regions was significant, however, that within striatal regions was not. This suggested that chronic-cocaine-induced cerebral ischemia was likely to initiate in cortical brain regions, and move into striatal regions only with more prolonged exposure. Indeed, after a longer period of cocaine exposure, e.g., 4 weeks, the microvascular density increased in NAc, as shown in Fig 3. VEGF expression also increased in NAc accordingly (Fig 4). This might support the clinical findings by Levine and Welch on cocaine-associated cerebrovascular complications in patients, i.e., five out of seven ischemic events were identified in cortical brain regions [34].

It’s interesting to notice that VEGF expression in the rat brain tissue was not necessarily co-localized with micro blood vessels. VEGF can be produced by many cell types, including vascular endothelial cells, platelets, astrocytes and macrophages [35–42]. It has been reported that hypoxia can trigger HIF-VEGF pathway activation in astrocytes [43]. Thus it is possible that vascular endothelial cells were not the only cells that contributed to the observed increase in VEGF expression following chronic cocaine treatment, and likely that astrocytes could have contributed as well. Overall, significant increases in microvascular density and VEGF expression were observed in rat brain tissue following chronic cocaine treatment. Increased microvascular density is a good indicator for angiogenesis, which requires endothelial cell proliferation and can result in cerebrovascular remodeling. Therefore, we interpret the cocaine-induced increases in the rat brain microvasculature (Fig 5) was a reflection of increased proliferation of endothelial cells, which led to cerebrovascular remodeling. To specifically quantify endothelial cell proliferation, the Edu incorporation assay may be used in our future studies.

HIF-1, the transcription factor for VEGF, can be activated by ischemia and hypoxic conditions. As expected, the expression of HIF-1α, a subunit of HIF-1, was significantly affected by chronic cocaine treatment, in a region-dependent manner. Significant changes in HIF-1α expression occurred within mPFC (Fig 7). Although large changes were observed in Ssc, no statistical significance was detected due to the large variability. HIF-1 plays critical roles in homeostasis. It helps to increase vascular density in hypoxic areas following ischemia or tumor growth [44]. We interpret the increased HIF-1α expression as a sign of the activation of this pathway following cocaine-induced vasoconstriction and ischemia, which triggered VEGF up-regulation and increased microvascular density, most markedly in cortical regions. Interestingly, significant increase in HIF-1α was only detected in mPFC (Fig 7), suggesting that physiological responses to restore damaged brain tissue/vasculature by chronic cocaine use may initiate in cortical regions. Alternatively it is also possible that cortical regions are more susceptible to cocaine-induced ischemia, which could explain why the physiological adaptations in these regions were seen earlier than that in striatal regions. Angiogenesis within the ischemic brain tissue can potentially restore local CBF and oxygen supply, and thus reverse the effects of cocaine. Indeed, therapeutic angiogenesis has been widely explored within the past twenty years as a potential approach to treat brain ischemic injury [45–48]. Even though it has been reported that angiogenesis adjacent to ischemic regions helped with longer survival of ischemic stroke patients [49], it is still under debate if therapeutic angiogenesis was effective, as it takes several days for the new blood vessels to grow, which is too long for neurons to survive following a stroke [50]. Also, many factors that promote angiogenesis increase vascular permeability (such as VEGF), which may cause undesired brain edema [51].

Chronic cocaine exposure led to NOS1 up-regulation, HIF-VEGF pathway activation and increases in microvascular density (or angiogenesis), all of which are physiological neuroadaptation responses associated with restoration of CBF under ischemic conditions. Promoting these physiological responses to restore CBF within ischemic area without causing brain edema is worth investigating, as it may lead to novel preventive or therapeutic solutions to cocaine-induced cerebral ischemia and stroke.

## Supporting information

S1 Fig Representative Western blot bands. HIF-1α expression following 2-week or 4-week cocaine treatment, in different brain regions.
(TIF)

S1 Dataset (XLSX)
