# Supplementary figures and images for: Chronic cocaine induces HIF-VEGF pathway activation along with angiogenesis in the brain
Source: PLoS One. 2017 Apr 27;12(4):e0175499. doi: 10.1371/journal.pone.0175499 (PMC5407832; doi:10.1371/journal.pone.0175499)

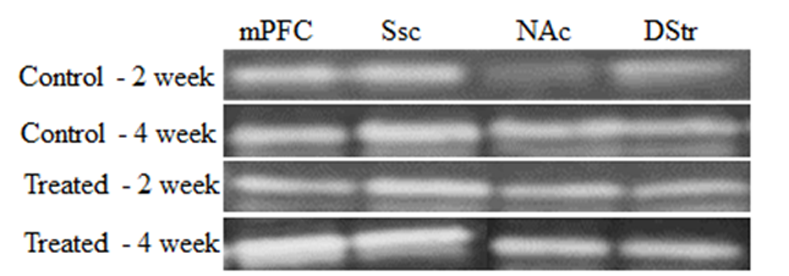

Supplement: S1 Fig — HIF-1α expression following 2-week or 4-week cocaine treatment, in different brain regions. (TIF) [file pone.0175499.s001.tif]
